# Interleukin-6 Receptor Polymorphism Is Prevalent in HIV-negative Castleman Disease and Is Associated with Increased Soluble Interleukin-6 Receptor Levels

**DOI:** 10.1371/journal.pone.0054610

**Published:** 2013-01-23

**Authors:** Katie Stone, Emily Woods, Susann M. Szmania, Owen W. Stephens, Tarun K. Garg, Bart Barlogie, John D. Shaughnessy, Brett Hall, Manjula Reddy, Antje Hoering, Emily Hansen, Frits van Rhee

**Affiliations:** 1 Myeloma Institute for Research and Therapy, University of Arkansas for Medical Sciences, Little Rock, Arkansas, United States of America; 2 Signal Genetics, LLC., New York, New York, United States of America; 3 Janssen Research and Development, Radnor, Pennsylvania, United States of America; 4 Cancer Research and Biostatistics, Seattle, Washington, United States of America; Baylor College of Medicine, United States of America

## Abstract

Multicentric Castleman Disease is largely driven by increased signaling in the pathway for the plasma cell growth factor interleukin-6. We hypothesized that interleukin-6/interleukin-6 receptor/gp130 polymorphisms contribute to increased interleukin-6 and/or other components of the interleukin-6 signaling pathway in HIV-negative Castleman Disease patients. The study group was composed of 58 patients and 50 healthy donors of a similar racial/ethnic profile. Of seven polymorphisms chosen for analysis, we observed an increased frequency between patients and controls of the minor allele of interleukin-6 receptor polymorphism rs4537545, which is in linkage disequilibrium with interleukin-6 receptor polymorphism rs2228145. Further, individuals possessing at least one copy of the minor allele of either polymorphism expressed higher levels of soluble interleukin-6 receptor. These elevated interleukin-6 receptor levels may contribute to increased interleukin-6 activity through the *trans*-signaling pathway. These data suggest that interleukin-6 receptor polymorphism may be a contributing factor in Castleman Disease, and further research is warranted.

## Introduction

Castleman Disease (CD) is a rare lymphoproliferative disorder first defined in 1954 [1]. CD may be divided into two major forms. Unicentric CD (UCD) is typically a slow-growing solitary mass occurring at a single anatomical site. Though the enlarging node may compress vital structures, UCD is rarely life-threatening, and surgical extirpation or localized radiotherapy is generally curative. Multicentric CD (MCD) affects multiple lymph node stations and often presents with lymphadenopathy, fever, weight loss, fatigue, edema, anemia and hypoalbuminemia [2]–[4]. In severe cases, patients may develop hepatosplenomegaly, massive ascites, pleural effusions, or organ failure, and MCD occasionally progresses to non-Hodgkin’s lymphoma. The clinical manifestations of MCD are largely driven by activation of the interleukin-6 (IL6) signaling cascade, which results in an acute phase reaction with elevated erythrocyte sedimentation rate, C-reactive protein (CRP), fibrinogen, thrombocytosis and hypergammaglobulinemia [2], [4], [5]. IL6 signaling enhances cell proliferation and differentiation, inhibits apoptosis, and leads to an increase in vascular endothelial growth factor and other cytokines [6], [7]. Conversely, blockade of IL6 signaling by monoclonal antibodies results in tumor regression and abrogation of symptoms [4].

Levels of IL6 have a significant impact on the prognosis of other IL6-related diseases and malignancies, including plasma cell dyscrasias (PCD).[8]–[10] Single nucleotide polymorphisms (SNPs) in the IL6 signaling pathway have been studied in acute phase reaction, auto-immune phenomena, and malignancies, and have been linked to elevated IL6 production.[11]–[14] Increased levels of IL6, along with soluble IL6R (sIL6R), have been detected in the synovial fluid of patients with rheumatoid arthritis [5], [6], [15]. IL6 promoter polymorphism may also be associated with an increased risk of PCD [14]. In MM, IL6-related polymorphism may be prognostic of survival [16], [17]. IL6 *trans*-signaling via sIL6R is also biologically important in that sIL6R allows cells that do not intrinsically express membrane-bound IL6R (mbIL6R) to be responsive to the effects of IL6.[5], [15], [18]–[20] The *IL6R* SNP rs2228145 is a functional polymorphism resulting in an asparagine to alanine substitution at the IL6R proteolytic cleavage site, just beyond the transmembrane domain of the IL6R protein. This SNP has been shown to significantly influence serum levels of sIL6R,[9], [17]–[19], [21], [22] and is thought to act by altering the efficiency of IL6R cleavage from the cell surface, which seems to be the dominant method of sIL6R generation [15], [18], [20], [23]. The *IL6R* SNP rs2228145 is in linkage disequilibrium with another *IL6R* SNP, rs4537545, and the minor allele of the rs4537545 SNP has been shown to account for nearly 20% of the variation in sIL6R levels [22].

We hypothesized that IL6/IL6R/gp130 polymorphisms contribute to increased IL6 and/or other components of the IL6 signaling pathway in CD patients. Seven well-studied SNPs in the *IL6* promoter (rs1800795, rs1800796, rs1800797), *IL6R* (rs2228145, rs4537545) and *gp130* (rs10940495, rs715180) were chosen for analysis. As CD is a prime example of an IL6-driven disease, we sought to determine whether CD patients express the minor allele of these IL6 pathway-related SNPs more frequently than healthy individuals, and assess what effect this may have on IL6/IL6R levels in CD patients versus healthy controls.

## Materials and Methods

### Ethics Statement

Research protocols were approved by the UAMS Institutional Review Board, and informed written consent was obtained from all participants before samples were collected, in accordance with the Declaration of Helsinki. The informed consent documents and consenting procedure were approved by the UAMS Institutional Review Board in accordance with UAMS and federal ethics guidelines.

### Study Samples

Peripheral blood mononuclear cells (PBMC) and serum were collected from 58 CD patients and 50 healthy donors (HD). HD had no history of malignancy or IL6-related disease. The CD patient cohort included both MCD (42) and UCD (16) patients, matched for age, sex and race/ethnicity to exclude SNP genotype bias (Table 1). All CD patients were HIV- and HHV8-negative by serology and quantitative PCR.

**Table 1:** Subject characteristics.

	CD	HD
Caucasian	44	43
African American	7	2
Hispanic	4	–
Median Age	42	35
Females	31	31
Males	27	19

The majority of both CD cases and HD controls are Caucasian (n = 44, 43 respectively) with a few subjects from other ethnicities. Both cohorts were also matched for age and gender.

### SNP Genotyping

DNA was extracted from PBMC using the DNeasy Blood and Tissue Kit (Qiagen, Valencia, CA). *IL6* promoter, *IL6R*, and *gp130* SNP genotypes were determined using TaqMan-based SNP genotyping assays (Applied Biosystems, Foster City, CA). PCR assays were performed in triplicate on the ABI Prism 7000 Sequence Detection System (SDS) instrument and analyzed using the Allelic Discrimination module of the SDS software (Applied Biosystems).

### sIL6R ELISA

Serum levels of sIL6R were determined via the Human IL6sR Quantikine ELISA Kit (R&D Systems, Minneapolis, MN) according to manufacturer specifications. Samples were assayed in duplicate. Data was acquired using SoftMax Pro software (Molecular Devices, Sunnyvale, CA), and analyzed in GraphPad Prism (GraphPad Software, La Jolla, CA).

### Meso-Scale Discovery Platform-based Panoptic IL6 Assay

Many capture or detection antibodies cannot recognize IL6 when in association with proteins in human serum such as alpha-2-macroglobulin, sIL6R, soluble gp130, complement proteins C3 and C4b, and albumin, thus skewing IL6 measurements. In an original report, Sehgal demonstrated that certain antibody pairs are able to recognize IL6 when in large molecular complexes that included IL6 associated proteins [24]. Given the discrepancies in methodologies for measuring IL6 and the number of variables that can affect antibody affinity for IL6 in serum, [25], [26] we developed a Panoptic IL6 assay for the measurement of IL6 in human serum. We investigated a large panel of commercially-available anti-IL6 antibodies for their ability to bind human recombinant IL6 in human serum. Top performing candidate capture and detection antibodies from our first generation screening were optimized in a sandwich ELISA format, and a second screening revealed the three best candidate capture antibodies. Immunoprecipitation experiments determined that the top three antibodies recognized and bound all known isoforms of IL6. Final antibody pairs were then developed on a Meso-Scale Discovery (MSD) platform, which resulted in a Panoptic IL6 assay for measuring IL6 in human serum. When compared to other validated IL6 platforms and antibodies, our final antibody pair of choice demonstrated a higher range of IL6 than other standard ELISA formats when measuring human IL6 in serum. (manuscript in preparation).

### Flow Cytometry

PBMC were labeled with mouse anti-human antibodies to determine levels of mbIL6R (CD126; R&D Systems) and gp130 (CD130; Becton Dickinson Biosciences, San Jose, CA) expression on T cells, monocytes, B cells, and natural killer cells. PBMC subset analysis was performed with FITC-conjugated antibodies specific for CD3, CD4, CD8, CD14, CD19, and CD56 (Becton Dickinson). Data was acquired on a FACSCalibur flow cytometer (Becton Dickinson) and analyzed using FCS Express analysis software (De Novo Software, Los Angeles, CA). Median fluorescence intensity (MFI) of the positive population (mbIL6R and gp130) was recorded for each cell type.

### Statistical Analysis

Significance of differences in minor allele frequencies (MinAF) between Castleman Disease (CD) patients and healthy donors (HD) was assessed by generating a z-score. The equation used was proposed by Schaid and Jacobsen [27]. This equation accounts for deviation from perfect Hardy Weinberg Equilibrium (HWE).
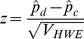
Where 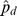 is the SNP’s major allele frequency (MajAF) in diseased subjects and 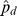 is the MajAF in controls.


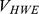
 is calculated as follows:
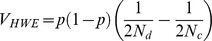
Where 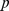 is the MajAF in all subjects, 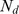 is the number of diseased subjects, and 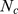 is the number of controls.

First, genotype frequencies were calculated for the entire cohort (CD + HD). Next, the MajAF and MinAF were calculated for each SNP. A Chi square was performed to determine whether the genotype frequencies were in HWE. The above formula was employed to generate the z-score for each SNP, which was then converted to a *p*-value utilizing GraphPad Prism (GraphPad Software).

Linkage disequilibrium (LD) was assessed utilizing the Online Encyclopedia for Genetic Epidemiology Studies’ CubeX Software (http://www.oege.org/software/cubex/). ELISA data was compared between CD patients and HD using Student’s *t* test.

## Results and Discussion

All SNPs tested were in Hardy Weinberg Equilibrium (HWE). LD was assessed for two SNPs in the *IL6* promoter region (rs1800795 and rs1800797) as well as two SNPs in the *IL6R* gene (rs2228145 and rs4537545), as they demonstrated similar allele frequencies. Consistent with previous reports, [14], [22] these two *IL6* promoter SNPs were found to be in LD (D’ = 1.0, r^2^ = 0.98), as were the two *IL6R* SNPs (D’ = 0.977, r^2^ = 0.77).

Observed allele frequencies for CD patients and HD are depicted in Table 2. A significant difference in the frequency of the minor T allele of *IL6R* SNP rs4537545 was observed between CD patients and HD (0.49 and 0.33, respectively; *p*<0.018). Similarly, the minor C allele of *IL6R* SNP rs2228145 was overexpressed in CD patients versus controls (0.42 vs. 0.30, *p*<0.068). While these two SNPs exhibit high LD, we observed minor discordance in a few subjects, which resulted in the greater *p*-value for the rs2228145 SNP. No significant differences in allele frequencies were detected in the other SNPs tested.

**Table 2:** SNP allele frequencies.

SNP	Gene	HD (n = 50)	CD (n = 58)	*p*-value
rs1800795	IL6	0.35	0.32	0.6410
rs1800797	IL6	0.35	0.32	0.6421
rs1800796	IL6	0.07	0.06	0.7660
rs4537545	IL6R	0.33	0.49	0.0175
rs2228145	IL6R	0.30	0.42	0.0679
rs10940495	gp130	0.32	0.21	0.0658
rs715180	gp130	0.09	0.09	1.0

Minor allele frequencies for each of the SNPs tested are depicted for HD and CD patients. *IL6* SNPs rs1800795 and rs1800797 are in LD as are the two *IL6R* SNPs. There is a significant difference between the minor allele frequencies of the *IL6R* SNP rs4537545 for HD and CD patients (*p* = 0.0175).

**Figure 1:**
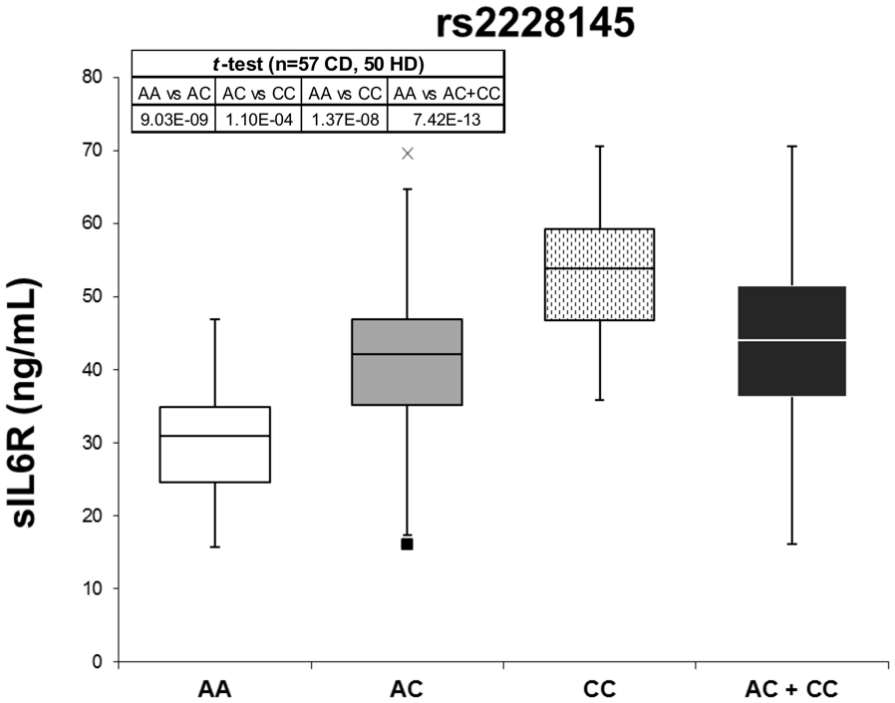
sIL6R levels. Levels of sIL6R in the serum of CD patients and HD were determined by ELISA. Subjects homozygous for the major allele demonstrate the lowest levels of sIL6R (white). Those who are heterozygous for the rs2228145 SNP exhibit increased levels of sIL6R in the serum (grey), while those who are homozygous for the minor allele (dots) produce the highest levels. Mean of the heterozygous and homozygous minor allele groups are also depicted (black). Min and max outliers for the heterozygous population are noted (black square and x, respectively). Differences in sIL6R levels among genotypes are highly significant.

Levels of sIL6R were measured in the serum (or plasma when serum was not available) of CD patients and controls by ELISA. Individuals possessing one or more copies of the minor C allele of *IL6R* SNP rs2228145 produced significantly higher levels of sIL6R (averages 43.98 ng/mL vs. 29.85 ng/mL, *p* = 7.42×10^−13^, Figure 1). Furthermore, individuals possessing at least one copy of the minor T allele of the *IL6R* SNP rs4537545 expressed higher levels of sIL6R than those homozygous for the major C allele (averages 42.84 ng/mL and 29.36 ng/mL, respectively, data not shown), and this difference was highly significant (*p* = 2.98×10^−12^). For both *IL6R* SNPs, this genotype-dependent difference in sIL6R levels was also observed in both the CD patient and control cohorts independently.

**Figure 2:**
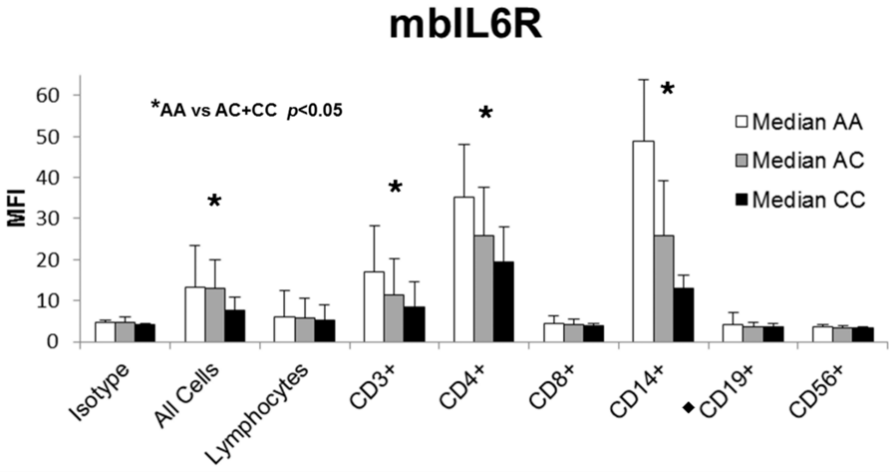
mbIL6R levels. Levels of mbIL6R for CD patients (n = 58) and HD (n = 50) were measured by flow cytometry. Subjects who are homozygous for the major allele exhibit the highest levels of mbIL6R (white). Levels are decreased at the cell surface of heterozygous subjects (grey) and lowest on minor allele-homozygous subjects (black). Reductions were significant on a subset of T cells and monocytes. CD19 data is omitted from 5 patients who received rituximab (black diamond). MFI: median fluorescence intensity.

**Figure 3:**
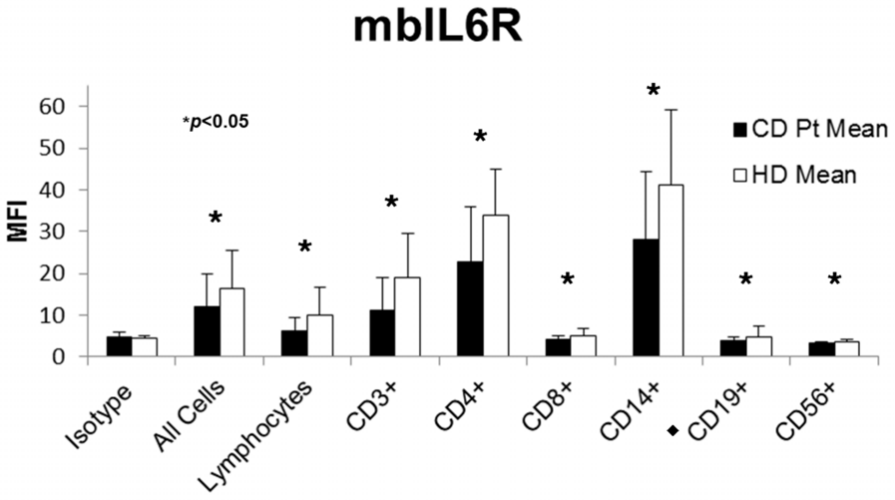
Mean mbIL6R levels. Levels of mbIL6R were measured by flow cytometry. Mean mbIL6R levels for HD (n = 50, white) are significantly greater than levels for CD patients (n = 58, black) on all cell types measured. CD19 data is omitted from 5 patients who received rituximab (black diamond).

Flow cytometry was performed to assess the levels of mbIL6R as well as gp130 on CD patient and HD PBMC. Antibodies to various lymphocyte and monocyte markers were incorporated to determine if changes in mbIL6R/gp130 levels were localized to a particular cell type. Subjects who were heterozygous for the *IL6R* SNP rs2228145 demonstrated decreased levels of mbIL6R at the cell surface as compared to those who were homozygous for the major A allele. Subjects homozygous for the minor C allele exhibited the lowest levels of mbIL6R. As was done with ELISA data, statistics were calculated by grouping heterozygous and minor-allele homozygous individuals together, and comparing to major-allele homozygous subjects. We observed a genotype-dependent difference in mbIL6R levels on most cell types studied (Figure 2). Similar results were obtained for the *IL6R* SNP rs4537545 (data not shown). When mean expression levels for CD patients were compared to HD, we observed that the overall mbIL6R expression is higher on PBMC from HD than CD patients (Figure 3).

Next, we assessed whether any correlations existed between these SNPs and IL6 levels in CD patients. Given the strong link between IL6 and CRP expression and the observation that patients with elevated serum CRP (>8 mg/L) can display apparently normal serum IL6 levels (< 10 pg/mL), we hypothesized that commonly used IL6 assays were not able to accurately quantify IL6 in all patients. We therefore employed the Panoptic IL6 assay due to its distinct ability to better quantify IL6 levels (complexed and non-complexed) in human blood (manuscript in preparation). Contrary to the observations made by Reich et al. and Rafiq et al., we did not see an association between genotype and IL6 levels [21], [22]. Variations among commercially-available IL6 assays in the ability to accurately detect IL6 levels could account for our inability to reproduce the results pertaining to IL6/SNP correlations found in others’ studies.

We found that CD patients express the minor allele of two SNPs in the *IL6R* gene (rs4537545 and rs2228145) at higher frequencies than do HD. Our data, similar to previous reports, [17], [18], [21], [22] showed that individuals possessing one or more copies of the minor allele of these *IL6R* SNPs exhibited increased levels of sIL6R, and these differences were highly significant. Similar findings were reported by our group in MM patients [17]. We studied the effect of *IL6R* SNPs on levels of mbIL6R, and observed that individuals who are heterozygous for the *IL6R* SNPs expressed reduced levels of IL6R at the cell surface, and those homozygous for the minor allele expressed the lowest levels of mbIL6R. This was significant for T cells and monocytes (*p*<0.05, Figure 2). Further, the mean expression of mbIL6R was greater in HD than CD patients. This data shows that CD patients express *IL6R* SNPs rs2228145 and rs4537545 more frequently, which leads to increased shedding of IL6R from the cell surface, increased levels of sIL6R in the serum, and presumably increased levels of IL6 signaling. This study identifies for the first time a potentially predisposing factor for MCD, namely the rs2228145 polymorphism in the *IL6R*, in this rare and complex disorder.

## References

[1] Castleman B, Towne VW (1954) Case records of the Massachusetts General Hospital; weekly clinicopathological exercises; founded by Richard C. Cabot. N Engl J Med 251: 396–400. 13194083 10.1056/NEJM195409022511008

[2] Castleman B, Iverson L, Menendez VP (1956) Localized mediastinal lymphnode hyperplasia resembling thymoma. Cancer 9: 822–830. 13356266 10.1002/1097-0142(195607/08)9:4<822::aid-cncr2820090430>3.0.co;2-4

[3] Peterson BA, Frizzera G (1993) Multicentric Castleman’s disease. Semin Oncol 20: 636–647. 8296200

[4] van Rhee F, Fayad L, Voorhees P, Furman R, Lonial S, et al (2010) Siltuximab, a novel anti-interleukin-6 monoclonal antibody, for Castleman’s disease. Journal of Clinical Oncology 28: 3701–3708. 20625121 10.1200/JCO.2009.27.2377

[5] Kishimoto T (2010) IL-6: from its discovery to clinical applications. International Immunology 22: 347–352. 20410258 10.1093/intimm/dxq030

[6] May LT, Santhanam U, Sehgal PB (1991) On the multimeric nature of natural human interleukin-6. Journal of Biological Chemistry 266: 9950–9955. 2033081

[7] Nakahara H, Song J, Sugimoto M, Hagihara K, Kishimoto T, et al (2003) Anti-interleukin-6 receptor antibody therapy reduces vascular endothelial growth factor production in rheumatoid arthritis. Arthritis & Rheumatism 48: 1521–1529. 12794819 10.1002/art.11143

[8] Bataille R, Jourdan M, Zhang XG, Klein B (1989) Serum levels of interleukin 6, a potent myeloma cell growth factor, as a reflect of disease severity in plasma cell dyscrasias. Journal of Clinical Investigation 84: 2008–2011. 2592570 10.1172/JCI114392 PMC304085

[9] Lagmay JP, London WB, Gross TG, Termuhlen A, Sullivan N, et al (2009) Prognostic significance of interleukin-6 single nucleotide polymorphism genotypes in neuroblastoma: rs1800795 (promoter) and rs8192284 (receptor). Clinical Cancer Research 15: 5234–5239. 19671870 10.1158/1078-0432.CCR-08-2953 PMC2740837

[10] Salgado R, Junius S, Benoy I, Van Dam P, Vermeulen P, et al (2003) Circulating interleukin-6 predicts survival in patients with metastatic breast cancer. International Journal of Cancer 103: 642–646. 12494472 10.1002/ijc.10833

[11] Fishman D, Faulds G, Jeffery R, Mohamed-Ali V, Yudkin JS, et al (1998) The effect of novel polymorphisms in the interleukin-6 (IL-6) gene on IL-6 transcription and plasma IL-6 levels, and an association with systemic-onset juvenile chronic arthritis. Journal of Clinical Investigation 102: 1369–1376. 9769329 10.1172/JCI2629 PMC508984

[12] Guerreiro CS, Ferreira P, Tavares L, Santos PM, Neves M, et al (2009) Fatty acids, IL6, and TNFalpha polymorphisms: an example of nutrigenetics in Crohn’s disease. American Journal of Gastroenterology 104: 2241–2249. 19550417 10.1038/ajg.2009.313

[13] Slattery ML, Wolff RK, Herrick JS, Caan BJ, Potter JD (2007) IL6 genotypes and colon and rectal cancer. Cancer Causes & Control 18: 1095–1105. 17694420 10.1007/s10552-007-9049-x PMC2442470

[14] Cozen W, Gebregziabher M, Conti DV, Van Den Berg DJ, Coetzee GA, et al (2006) Interleukin-6-related genotypes, body mass index, and risk of multiple myeloma and plasmacytoma. Cancer Epidemiology, Biomarkers & Prevention 15: 2285–2291. 10.1158/1055-9965.EPI-06-0446 17119059

[15] Peake NJ, Khawaja K, Myers A, Nowell MA, Jones SA, et al (2006) Interleukin-6 signalling in juvenile idiopathic arthritis is limited by proteolytically cleaved soluble interleukin-6 receptor. Rheumatology 45: 1485–1489. 16690760 10.1093/rheumatology/kel154

[16] Mazur G, Bogunia-Kubik K, Wrobel T, Karabon L, Polak M, et al (2005) IL-6 and IL-10 promoter gene polymorphisms do not associate with the susceptibility for multiple myeloma. Immunology Letters 96: 241–246. 15585329 10.1016/j.imlet.2004.08.015

[17] Stephens OW, Zhang Q, Qu P, Zhou Y, Chavan S, et al (2012) An intermediate-risk multiple myeloma subgroup is defined by sIL-6r: levels synergistically increase with incidence of SNP rs2228145 and 1q21 amplification. Blood 119: 503–512. 22072558 10.1182/blood-2011-07-367052 PMC3257015

[18] Galicia JC, Tai H, Komatsu Y, Shimada Y, Akazawa K, et al (2004) Polymorphisms in the IL-6 receptor (IL-6R) gene: strong evidence that serum levels of soluble IL-6R are genetically influenced. Genes & Immunity 5: 513–516. 15306846 10.1038/sj.gene.6364120

[19] Mullberg J, Oberthur W, Lottspeich F, Mehl E, Dittrich E, et al (1994) The soluble human IL-6 receptor. Mutational characterization of the proteolytic cleavage site. Journal of Immunology 152: 4958–4968. 8176214

[20] Jones SA, Horiuchi S, Topley N, Yamamoto N, Fuller GM (2001) The soluble interleukin 6 receptor: mechanisms of production and implications in disease. FASEB Journal 15: 43–58. 11149892 10.1096/fj.99-1003rev

[21] Reich D, Patterson N, Ramesh V, De Jager PL, McDonald GJ, et al (2007) Admixture mapping of an allele affecting interleukin 6 soluble receptor and interleukin 6 levels. American Journal of Human Genetics 80: 716–726. 17357077 10.1086/513206 PMC1852718

[22] Rafiq S, Frayling TM, Murray A, Hurst A, Stevens K, et al (2007) A common variant of the interleukin 6 receptor (IL-6r) gene increases IL-6r and IL-6 levels, without other inflammatory effects. Genes & Immunity 8: 552–559. 17671508 10.1038/sj.gene.6364414 PMC2668154

[23] Scheller J, Rose-John S (2006) Interleukin-6 and its receptor: from bench to bedside. Medical Microbiology & Immunology 195: 173–183. 16741736 10.1007/s00430-006-0019-9

[24] Sehgal PB (1996) Interleukin-6-type cytokines in vivo: regulated bioavailability. Proceedings of the Society for Experimental Biology & Medicine 213: 238–247. 8985307 10.3181/00379727-213-44055

[25] Ledur A, Fitting C, David B, Hamberger C, Cavaillon JM (1995) Variable estimates of cytokine levels produced by commercial ELISA kits: results using international cytokine standards. Journal of Immunological Methods 186: 171–179. 7594617 10.1016/0022-1759(95)00184-c

[26] Krakauer T (1998) Variability in the sensitivity of nine enzyme-linked immunosorbant assays (ELISAs) in the measurement of human interleukin 6. J Immunol Methods 219: 161–167. 9831397 10.1016/s0022-1759(98)00138-0

[27] Schaid DJ, Jacobsen SJ (1999) Biased tests of association: comparisons of allele frequencies when departing from Hardy-Weinberg proportions. American Journal of Epidemiology 149: 706–711. 10206619 10.1093/oxfordjournals.aje.a009878

